# High-Speed Lateral Flow Strategy for a Fast Biosensing with an Improved Selectivity and Binding Affinity

**DOI:** 10.3390/s18051507

**Published:** 2018-05-10

**Authors:** Dong Guk Cho, Haneul Yoo, Haein Lee, Yeol Kyo Choi, Minju Lee, Dong June Ahn, Seunghun Hong

**Affiliations:** 1 Department of Physics and Astronomy and Institute of Applied Physics, Seoul National University, Seoul 08826, Korea; eastmania@snu.ac.kr (D.G.C.); h30461@gmail.com (H.Y.); m201320376@snu.ac.kr (M.L.); 2 Department of Chemical & Biological Engineering, KU-KIST Graduate School of Converging Science & Technology, Korea University, Seoul 02841, Korea; godls5933@naver.com (H.L.); kyo_choi@korea.ac.kr (Y.K.C.)

**Keywords:** lateral flow, rotating disk, reaction speed, binding affinity, selectivity

## Abstract

We report a high-speed lateral flow strategy for a fast biosensing with an improved selectivity and binding affinity even under harsh conditions. In this strategy, biosensors were fixed at a location away from the center of a round shape disk, and the disk was rotated to create the lateral flow of a target solution on the biosensors during the sensing measurements. Experimental results using the strategy showed high reaction speeds, high binding affinity, and low nonspecific adsorptions of target molecules to biosensors. Furthermore, binding affinity between target molecules and sensing molecules was enhanced even in harsh conditions such as low pH and low ionic strength conditions. These results show that the strategy can improve the performance of conventional biosensors by generating high-speed lateral flows on a biosensor surface. Therefore, our strategy can be utilized as a simple but powerful tool for versatile bio and medical applications.

## 1. Introduction

The delivery of target molecules by liquid flows has been often utilized in various biosensing systems. For example, a rapid kit based on a lateral flow assay used a sample flow driven by the capillary force of a liquid sample [1]. In the kit, a liquid sample containing the analyte of interest was loaded onto one end of the kit, and the sample flowed through various zones of receptor-coated strips. As another example, in a lab-on-chip system, the analyte solutions flow through micro channels, which conduct a series of reaction processes, including the pretreatment of samples and the binding reaction between the targets and their receptors [2]. In these examples, the lateral flow of the analyte solutions is mostly used for the delivery of the analyte solution to the desired location of the sensor.

Meanwhile, the performances of biosensing systems, including the examples above, are often limited by several factors. For example, the diffusivity of the target molecules could affect the detection limit and speed of a biosensor [3]. Moreover, the non-specific binding of molecules could lead to a decrease in the sensitivity of biosensors by increasing the background noise of sensor signals [4]. These factors could result in fundamental limitations on conventional biosensing systems. Thus, various techniques have been suggested to overcome the fundamental limitations of biosensing systems. For example, motion-based approaches using microstructures such as magnetic particles and micromotors have been used to enhance the detection speeds of biosensors, where target molecules increase their mass transport rates to a sensing surface with the assistance of the microstructures [5,6]. However, the small size of microstructures may lead to difficulties in the fabrication and control of the structures. Additionally, a rotating disk system has been developed to overcome the detection speed limit of conventional biosensor systems [7,8,9]. In this case, sensors are fixed at the center of a rotating disk and rotated at a high speed. The rotation of the disk could increase the vertical influx of target molecules toward the sensor surface. These methods can be utilized to improve detection speed, but it is difficult to enhance the sensitivity and selectivity of a biosensor. Recently, the lateral fluid flows of an analyte solution have been utilized to enhance the selectivity of a biosensor by removing nonspecifically bound molecules from the capture surface of a biosensor [10,11]. In this method, alternating current (AC) electric fields are utilized to drive the lateral fluid flows of the analyte solution on the capture surface. However, this method requires a specifically patterned structure of the biosensor and precise control of the AC electric fields in order to generate lateral flows on the sensor, which makes it expensive and tricky.

Herein, we report a high-speed lateral flow strategy for a fast biosensing with an improved selectivity and binding affinity even under harsh conditions. In this strategy, to create the high-speed lateral flow (flow speed ~120 mm/s) of the target solution with respect to the biosensor, the biosensor is fixed at a location away from the center of a rotating disk, and the disk is rotated at a rather high speed (~150 rpm) during the sensing measurement. We found that the outermost biosensors, which were 15 mm from the center of the disk, showed a 60% improvement in the binding rate of the target molecules compared to the innermost biosensors at the center of the disk, which directly indicates the importance of the lateral flow speeds on biosensor characteristics. Interestingly, weakly bound molecules on the biosensor surface were removed from the laterally moving solution by shear forces, which, in effect, removed nonspecifically bound molecules and improved the selectivity of the biosensors. Furthermore, we found that the binding affinity between target molecules and sensing molecules was enhanced even under very harsh conditions, such as an acidic pH 3 and a low ionic concentration of 3 mM, which could be important in improving the detection limit of biosensors in real samples. These results show that the performance of conventional biosensors could be improved simply by moving the sample solution laterally at a high speed, and thus, our lateral flow strategy could be a simple but powerful tool for versatile bio and medical applications.

## 2. Materials and Methods

### 2.1. High-Speed Lateral Flow-Based Biosensing System Using a Rotating Disk

**Figure 1:**
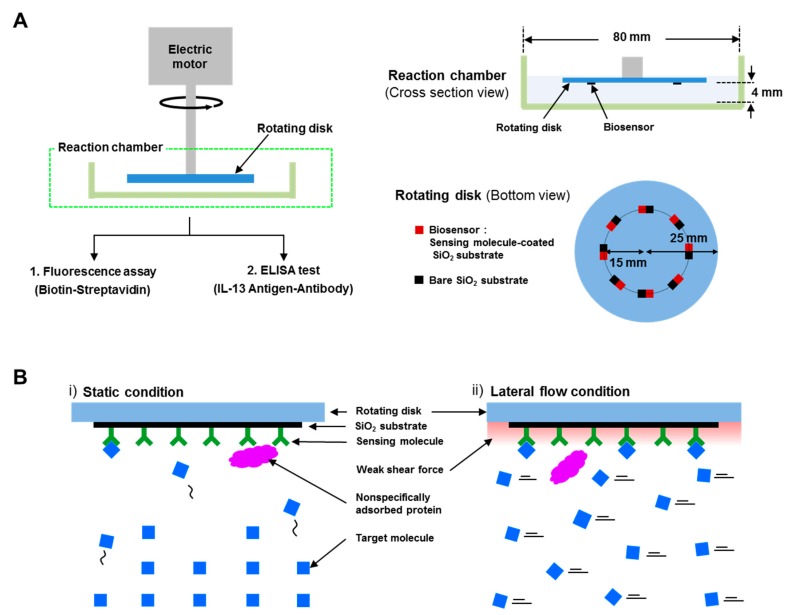
(**A**) Schematic diagram of a high-speed lateral flow biosensing system; (**B**) Schematic diagrams depicting the motion of molecules near the surface of a biosensor under (**i**) static conditions and (**ii**) lateral flow conditions.

Figure 1A shows the schematic diagram of a high-speed lateral flow biosensing system. The system consisted of a reaction chamber and a rotating disk, which was connected to an electrical motor via a rotating bar. The electrical motor (DAIHAN Scientific Co., Ltd., WiseStir, Seoul, Korea) had a rotating speed range from 0 and 3000 rpm with an accuracy of 1%. The slew rate of the motor was ~100,000 rpm/s, which enabled us to obtain the desired rotating rate (0–300 rpm) instantaneously. The reaction chamber, which contained the target solution, was a commercial petri dish with a diameter of 80 mm. The rotating disk had a dimeter of 25 mm, and biosensors and bare SiO_2_ substrates were attached on the disk side by side in regions 15 mm from the center of the disk. The distance between the rotating disk and the bottom of the reaction chamber was 4 mm. The system was designed to generate high-speed lateral flow of the target solution on the biosensors and bare SiO_2_ substrates, when the disk was rotated in the target solution. The lateral flow speed of the target solution was simply calculated by multiplying the angular velocity of the rotating disk by a distance from the center of the disk to the position of the biosensor. As a proof of concept, we used a sensing molecule-coated SiO_2_ substrate as a biosensor, which was considered to be the sensing part of conventional biosensors. Because conventional biosensors use a sensing molecule-coated surface to capture target molecules, sensing molecule-coated SiO_2_ substrates were chosen as a simple biosensor in the system. Specifically, we used biotin-coated SiO_2_ substrates to detect streptavidin proteins and interleukin-13 (IL-13) antibody-coated SiO_2_ substrates to detect IL-13 antigen proteins. Bare SiO_2_ substrates were also used to analyze the non-specific bindings of target proteins. In this study, we conducted detection experiments using a fluorescence assay and an enzyme-linked immunosorbent assay (ELISA), which are widely used for biosensing systems due to their high reliability. It should also be noted that since the system can easily generate lateral flows in the target solution just by the arrangement of biosensor positions on a rotating disk, it could be utilized in various biosensing systems.

Figure 1B shows the plausible motions of target proteins near the surface of biosensors under (i) static conditions and (ii) lateral flow conditions. Under the static conditions in Figure 1(Bi), the target proteins can move and bind to their sensing molecules on a biosensor surface by diffusive motions. On the other hand, under the lateral flow conditions in Figure 1(Bii), a rotating disk generates the lateral flows of the target solution on the biosensor surface, delivering the target proteins continuously to the biosensor surface. Moreover, the lateral flows generate a weak shear force on the biosensor surface, which may remove weakly bound molecules on the biosensor surface. It should be mentioned that since the system for our method only requires a small-sized electrical motor and it can be applied to virtually all biosensors, it could be utilized as a portable tool to improve the performance of nanoscale biosensors for practical applications.

### 2.2. Test on Fluorescence Assay

Our lateral flow biosensing system was tested in a fluorescence assay. Streptavidin molecules labeled with fluorescein isothiocyanate dyes (FITC-labeled streptavidin) and biotinylated SiO_2_ substrates were utilized as target molecules and biosensors, respectively. The biotinylated SiO_2_ substrates were prepared using a biotinylation method reported previously [12]. Briefly, cleaned SiO_2_ substrates (3 mm × 3 mm) were immersed in a 3-aminopropyl-triethoxysilane (APTES, Sigma-Aldrich. Inc., 440140, Seoul, Korea) solution (APTES:toluene = 1:100) at 70 °C for 12 h. The APTES-coated substrates were transferred to a 2 mg/mL biotin N-hydroxysuccinimide ester (biotin-NHS, Sigma Aldrich. Inc., H1759, Seoul, Korea) solution for 1 h at room temperature for the biotinylation of biotin-NHS molecules with APTES molecules on the substrates. Then, the biotinylated substrates were washed three times with a phosphate-buffered saline (PBS) solution to remove loosely bound biotin-NHS molecules on the substrates. In order to block nonspecific binding sites, the biotinylated substrates and bare SiO_2_ substrate were incubated in a 2 wt % bovine serum albumin (BSA, Sigma Aldrich. Inc., A2153, Seoul, Korea) solution for 1 h, and then the prepared biotinylated and bare substrates were fixed on a rotating disk using double-sided adhesive tape. Next, we prepared 1 ng/mL streptavidin target solutions by dissolving FITC-labeled streptavidin powders (Sigma Aldrich. Inc., S3762, Seoul, Korea) in 2 wt % BSA-diluted PBS solution. For the sensing experiments of streptavidin molecules, the rotating disk including the biotinylated substrates was immersed in the target solution, and the disk was rotated to generate the static and lateral flow of the target solution for 90 min. The rotating speeds of the disk were adjusted to achieve the desired lateral flow speeds of the target solution on the biotinylated substrates (0 mm/s for static conditions and 120 mm/s for lateral flow conditions). After the sensing procedures, the disk was gently washed three times with a PBS solution, and the fluorescence images of the substrates were taken by a fluorescence microscope (Nikon Instruments Inc., Eclipse te2000-u, New York, NY, USA) in a PBS solution. A MetaMorph image program (Molecular Device. Inc., Version 7.7, San Jose, CA, USA) was used to obtain the average fluorescence intensities (100 µm × 100 µm) at each substrate. All experiments were repeated at least three times with eight samples in each experiment.

### 2.3. Test on ELISA 

ELISA is a detection method that is widely used in biosensing systems. In particular, the signal amplification step by enzyme reactions in the method allows the method to be versatile in different biosensing systems requiring highly sensitive detection of target molecules [13,14,15]. We also tested our method on conventional ELISA using commercial ELISA test kits (Thermo Fisher Scientific, Ready-Set-Go!, Waltham, MA, USA). First, IL-13 antibodies were coated on SiO_2_ substrates using a previously described protein coating method [16]. Briefly, cleaned SiO_2_ substrates were immersed in a 3-Mercaptopropyl trimethoxysilane (MTS, Sigma Aldrich. Inc., 175617, Seoul, Korea) solution (MTS:toluene = 1:50) at room temperature for 2 h. The substrates were rinsed by toluene and dried with the stream of nitrogen gases. Then, the MTS-coated substrates were immersed in a 4-Maleimidobutyric acid N-hydroxysuccinimide ester (GMBS, Sigma Aldrich. Inc., 63175, Seoul, Korea) solution (2 mM GMBS in DI water) for 2 h at room temperature, and the substrates were washed three times with DI water. The GMBS-coated substrates were transferred to a 96-well plate, and the substrates were incubated with the 100 µL/well of IL-13 antibody solutions for 12 h at 4 °C. After the incubation, the substrates were washed three times with PBS solution to remove loosely bound IL-13 antibodies on the substrates. In order to block nonspecific binding sites, the IL-13 antibody-coated substrates were incubated in a 2 wt % bovine serum albumin (BSA, Sigma Aldrich. Inc., A2153, Seoul, Korea) solution for 1 h. These IL-13 antibody-coated substrates were used as biosensors for capturing IL-13 antigens. The substrates were fixed onto a rotating disk using double-sided adhesive tape. Next, the target solutions of IL-13 antigens were prepared in 2 wt % BSA solutions with concentration ranges from 0.01 to 100 ng/mL. For the sensing procedures of IL-13 antigens, the rotating disk including the IL-13 antibody-coated substrates was immersed in IL-13 antigen solutions for 2 h under static and lateral flow conditions. After the sensing procedures, the reacted substrates were collected from the disk and were placed in a new 96-well plate for the remaining steps of ELISA. In order to form the sandwich structure of the 1st antibody–antigen–2nd antibody, the substrates were incubated in 100 μL of biotin-linked 2nd antibody solutions for 1 h at room temperature, followed by rinsing 5 times with a wash buffer (1 × PBS with 0.05% Tween-20). Then, the substrates were treated with avidin-linked horseradish peroxidase (HRP) solution and tetramethylbenzidine (TMB) solution for 30 min and 15 min, respectively. Then, 1 M H_3_PO_4_ solutions were applied to stop the enzyme reactions of HRP with TMB. Reacted solutions were transferred to a new 96-well plate. Finally, the absorbance values of the transferred solutions were measured using a multiple plate reader (Perkin Elmer. Inc., Victor3, Waltham, MA, USA) at 450 nm. The experiments were repeated at least three times, with eight samples in each experiment.

### 2.4. Simulation

A commercial finite element method package (COMSOL Multiphysics) was used for the simulation of fluidic flows and shear rates in the reaction chamber. A two-dimensional cross-section structure, including a rotating disk, a target solution and a reaction chamber, was considered for the simulation. A rotational symmetric boundary condition was applied. Three modules of a laminar flow interface, a transport interface and a reaction interface were utilized. To calculate the shear force of a target solution for bound target molecules in the 15 mm region of the disk, we attached a 10 nm-sized particle in the region and simulated the shear rate of the solution. The shear force near the particle was estimated by multiplying the shear rate by the viscosity of the solution. 

## 3. Results and Discussion

### 3.1. Enhanced Biosensing Speed and Improved Binding Affinity of Interacting Proteins in a High-Speed Lateral Flow System

**Figure 2:**
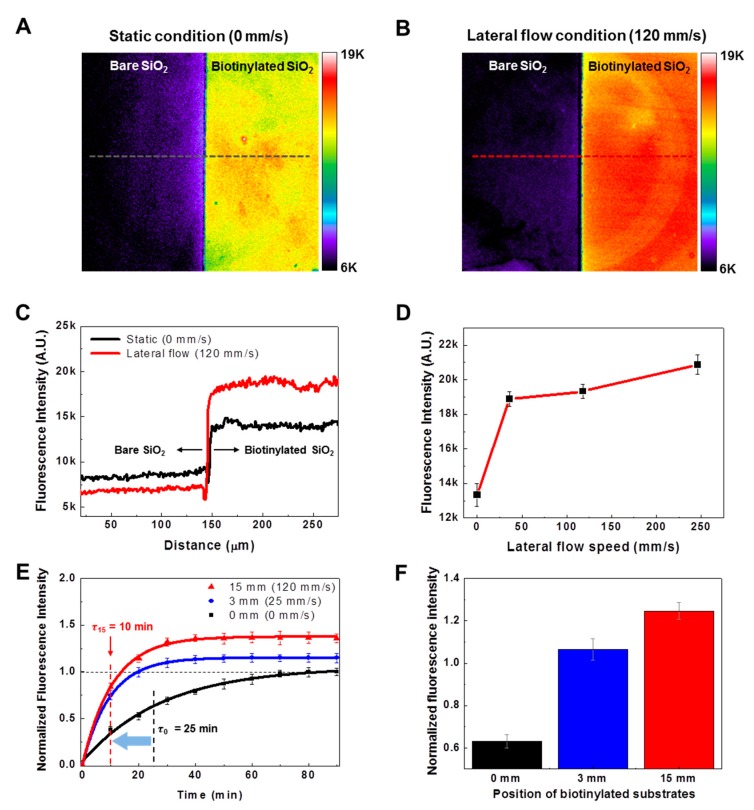
Effect of lateral flows on binding reactions between a biotinylated SiO_2_ substrate and streptavidin molecules: (**A**) Fluorescence images of bare and biotinylated SiO_2_ substrates after reaction with FITC-labeled streptavidin molecules under static conditions; (**B**) Fluorescence images of bare and biotinylated SiO_2_ substrates after reaction with FITC-labeled streptavidin molecules under lateral flow conditions; (**C**) Comparison of amount of bound streptavidin molecules on a bare and a biotinylated SiO_2_ substrate with and without lateral flow (120 mm/s). The fluorescence profiles were obtained from the fluorescence images of Figure 1A,B; (**D**) Fluorescence intensities of biotinylated substrates after reaction with FITC-labeled streptavidin molecules under lateral flow speeds of 0, 30, 120 and 250 mm/s; (**E**) Real-time fluorescence measurement data obtained from biotinylated substrates during reaction with FITC-labeled streptavidin molecules. The biotinylated substrates were fixed to a rotating disk at 0, 3 and 15 mm from the center of the disk.; (**F**) Fluorescence intensities of bound FITC-labeled streptavidin molecules on biotinylated substrates attached at different positions on the rotating disk. The fluorescence intensities were obtained at the reaction time of 25 min in Figure 1E.

Figure 2A shows the fluorescence image of a bare SiO_2_ substrate (left) and a biotinylated SiO_2_ substrate (right) after reactions with FITC-labeled streptavidin molecules under static conditions. The reactions were conducted for 90 min, and then the fluorescence images of both substrates were obtained in PBS solution by a fluorescence microscope. The biotinylated substrate exhibited much brighter fluorescence intensities than the bare SiO_2_ substrate. Since fluorescence intensities are correlated with the amount of bound FITC-labeled streptavidin molecules on the biotinylated substrates, the results indicate that the biotinylated substrate could effectively capture streptavidin molecules, as we expected.

We also conducted a reaction experiment under lateral flow conditions. Figure 2B shows the fluorescence image of bare and biotinylated SiO_2_ substrates after reaction with streptavidin molecules for 90 min. In the experiment, we fixed the biotinylated substrates 15 mm away from the center of the rotating disk, and the disk was rotated at 150 rpm to achieve a lateral flow speed of ~120 mm/s in a target solution on the biotinylated substrate. Note that the biotinylated substrate under lateral flow conditions exhibited higher fluorescence intensities than that under static conditions. This indicates that the lateral flows of target solutions could increase the binding events between target molecules and their sensing molecules on a substrate.

Figure 2C shows the fluorescence intensity profiles of the bare and the biotinylated SiO_2_ substrates under static (red line) and lateral (black line) flow conditions. The profiles were obtained along a black-dotted line in Figure 2A and a red-dotted line in Figure 2B. The average fluorescence intensities of the biotinylated substrate are about 14,100 under static conditions and about 18,570 under lateral flow conditions. These results indicate a 31.7% increase of bound streptavidin molecules on the biotinylated substrate simply as a result of flowing the target solution laterally. Interestingly, the average fluorescence intensities of the bare SiO_2_ substrates are ~8440 under static conditions and ~6860 under lateral flow conditions, which indicates an 18.7% decrease of bound target molecules as a result of the lateral flow of the target solution on the bare SiO_2_ substrates. These results indicate that the lateral flow of the target solution can not only increase the specific bindings on the biotinylated substrate but also decrease the nonspecific adsorptions on the bare SiO_2_ surface.

Figure 2D shows the fluorescence intensity graph of biotinylated substrates after reactions with FITC-labeled streptavidin molecules under the lateral flows with the different speeds. To achieve the lateral flow speeds of 0, 30, 120 and 250 mm/s, we fixed the biotinylated substrates at 15 mm from the center of the rotating disk, and rotated the disk at 0, 40, 150 and 300 rpm. The graph shows the increase of fluorescence intensities as the lateral flow speed increases. Previously, it was reported that the binding speed of target molecules on a sensing substrate in a rotating disk system was improved as the rotating speed of the disk increased [17,18], which was consistent with our results. In the previous works, the rotation of the disk improved the mass transfer rate of the target molecules to the sensing substrate, thereby increasing the amount of bound target molecules on the sensing substrate. In our strategy, the sensing substrates were attached to the rotating disk at 15 mm, where high lateral flows with 120 mm/s were generated. Therefore, the effects of lateral flows on the binding kinetics between streptavidin and a biotinylated substrate also need to be considered.

To investigate only the effects of the lateral flows of a target solution on binding reactions between streptavidin and a biotinylated substrate, we performed binding reactions by changing the biotinylated substrate positions (0, 3 and 15 mm) on a rotating disk, but without changing the rotating speed of the disk. Figure 2E shows the real-time fluorescence measurement data obtained from biotinylated SiO_2_ substrates during reactions with FITC fluorescent-labeled streptavidin molecules. In this experiment, the biotinylated substrates were fixed at 0, 3 and 15 mm from the center of the disk, and the disk was rotated at 150 rpm to achieve the lateral flow of the target solution with speeds of 0, 25 and 120 mm/s, respectively. To evaluate the sensing speed, the data were fitted by the first-order rate equation:

(1) $$A\left(t\right)=A_{max}\left[1-e^{-\frac{t}{\tau }}\right]$$

where, *A*(*t*), *A_max_*, and τ indicate the amount of bound target molecules on a biosensor at time t, the maximum amount of bound target molecules on the biosensor, and the characteristic time constant of the reaction, respectively [19]. By fitting the data, we were able to estimate the time constants τ of the biotinylated substrates at 0, 3 and 15 mm as τ_0_ ~ 25 min, τ_3_ ~ 10 min and τ_15_ ~ 10 min, respectively. Note that the time constants of the substrates at 3 and 15 mm from the disk center were reduced by 60% compared with those of the substrates at the center, although the substrates at the disk center should have a larger vertical flow. It was reported previously that if there are convective flows toward the sensing substrate, the local velocity of flows is zero at the center of the substrate [20]. Presumably, the zero velocity at the center of the rotating disk could reduce the rate of reactions due to the mass transfer rate of target molecules attenuated by the zero-flow velocity. On the other hand, the flow velocities in regions other than the center of the disk are not zero, due to the lateral flows of the target solution. Therefore, the sensing substrate at 3 and 15 mm showed higher sensing speed than the sensing substrate at 0 mm. Moreover, it should be noted that the characteristic time of a streptavidin-biotin binding reaction was calculated from the saturation time of the reactions in order to remove unexpected errors resulting from fast reaction speeds at an initial stage. Since our results show the effect of lateral flows on the sensing speed of a biosensing system, the enhanced sensing speeds can also be achieved in various biosensing systems by the lateral flow strategy.

Figure 2F shows the fluorescence intensities of biotinylated substrates at different distances from the center of a rotating disk at a reaction time of 25 min. The fluorescence intensities of the biotinylated substrates at 0, 3 and 15 mm from the disk center were 0.632 ± 0.03, 1.064 ± 0.05 and 1.246 ± 0.04, respectively. Note that the substrates with a larger lateral flow exhibited a larger binding of target molecules, even with the same reaction time, indicating enhanced binding events of target molecules as a result of lateral solution flow. We simulated the lateral and vertical flow speeds of the target solution (Supplementary Figure S1). We found that the lateral flow speeds were proportional to the distances from the center of the disk, while the vertical flow speeds remained almost unchanged. Previously, it was reported that the amount of bound target molecules could be increased by increasing the collision frequency between target molecules and a sensing substrate [21]. Presumably, in our works, the larger lateral flow speed of a target solution on the sensing substrate at 15 mm could increase the collision frequency of target molecules to the sensing substrate more than that at 3 mm. Therefore, higher fluorescence intensities on the sensing substrate at 15 mm were observed than on the sensing substrate at 3 mm. We also simulated the binding events between target molecules and sensing substrates located at 0, 3, 9 and 15 mm of the disk (Supplementary Figure S2) and obtained simulation results consistent with the experimental results in Figure 2F. The simulation results also show the turbulent flows of a target solution over 15 mm from the disk center, indicating that 15 mm from the disk center is the outermost position at which the sensing substrate can be located.

**Figure 3:**
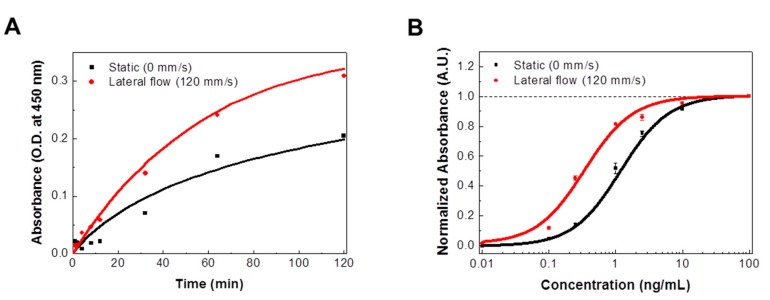
Effects of lateral flow conditions on IL-13 antibody-antigen reactions: (**A**) Absorbance values of bound IL-13 antigens to IL-13 antibody-coated substrates with or without lateral flows (120 mm/s); (**B**) Dose-response of IL-13 antibody-coated substrates to IL-13 antigens with or without lateral flows (120 mm/s).

Figure 3A shows the absorbance values of bound IL-13 antigens to IL-13 antibody-coated substrate under static (black dot) and lateral (red dot) flow conditions. We loaded eight IL-13 antibody-coated substrates on the rotating disk and performed the reaction experiments in IL-13 antigen target solution with and without lateral flows (120 mm/s). During the reaction process, each reacted substrate was collected from the disk at various reaction times, and the absorbance values of the reacted substrates were obtained by using an ELISA method (see the Materials and Methods section). The absorbance data points were fitted using Equation (1). The graph shows that the responses under lateral flow conditions exhibited higher absorbance values than those under static conditions. Since absorbance values indicate the amount of bound IL-13 antigens to the antibody-coated substrates, the results show that the amount of bound IL-13 antigens increased as a result of the lateral flows of the target solution even in an immune protein reaction. Based on these results, we could expect that the lateral flow strategy could be applied in various sensing applications simply by changing the sensing substrates on a rotating disk.

Figure 3B shows the dose-response of IL-13 antibody-coated substrates to IL-13 antigens under static and lateral flow conditions. The reaction experiments were conducted in IL-13 antigen solution for 2 h with the concentration range of the IL-13 antigen solution from 0.01 to 100 ng/mL. The experimental data points were fitted using Hill’s equation [22]. Under static conditions, the equilibrium dissociation constant K of IL-13 was about 1.18 ± 0.1 ng/mL, which was consistent with the previously reported value [23]. However, under lateral flow conditions, the constant K of IL-13 was about 0.34 ± 0.08 ng/mL, which was three times lower than that observed under static conditions. This indicates an enhanced binding affinity in an immune protein reaction as a result of the lateral flow of the target solution, which has not been reported before. We also estimated the limit of detection (LOD) from Figure 3B. The limit of detection is defined as the concentration yielding a signal equal to the blank signal plus three times its standard deviation [17,18]. Based on the results of IL-13 tests, LODs of 69 ± 1 pg/mL and 21 ± 0.3 pg/mL under static and lateral flow conditions, respectively, were obtained. These results clearly show the enhanced LOD under the lateral flow condition.

### 3.2. Reduced Nonspecific Adsorptions 

**Figure 4:**
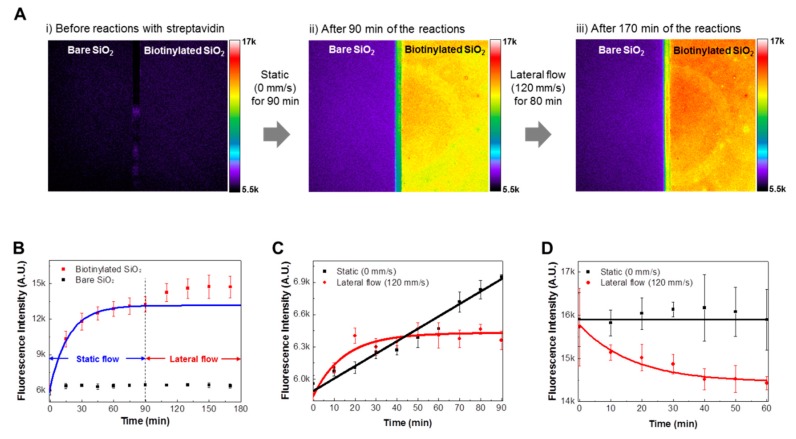
Reduced nonspecific adsorptions under lateral flow conditions: (**A**) (**i**) Fluorescence image of bare and biotinylated substrate before reaction with FITC-labeled streptavidin molecules, (**ii**) Fluorescence image of both substrates after 90 min reaction under static conditions, (**iii**) Fluorescence image of both substrates after 170 min reaction under static and lateral flow conditions. The experiment was performed under static conditions for 90 min, followed by under lateral flow conditions for 80 min; (**B**) Real-time response curves of the bare (black dot) and the biotinylated (red dot) substrates to FITC-labeled streptavidin molecules. The experiments were conducted under the experimental conditions of Figure 4A; (**C**) Nonspecific binding reaction curves of streptavidin molecules to bare SiO_2_ substrates under static (black dot) and lateral (red dot) flow conditions; (**D**) Dissociation curves of nonspecifically bound streptavidin molecules to bare SiO_2_ substrates under static (black dot) and lateral (red dot) flow conditions.

Figure 4A shows the fluorescence images of bare and biotinylated SiO_2_ substrates (i) before reactions with FITC-labeled streptavidin molecules, (ii) after reaction under static conditions for 90 min, and (iii) after reaction under static and lateral flow conditions for 170 min. The reaction experiments were sequentially performed under a static condition for 90 min, and then under a lateral flow condition for 80 min. Before reactions with FITC-labeled streptavidin molecules (Figure 4(Ai)), only background fluorescence intensities were measured on both the bare and the biotinylated substrates. After reaction under static conditions (Figure 4(Aii)), clear differences in fluorescence intensities between bare and biotinylated substrates were observed. Interestingly, after reaction under lateral flow conditions (Figure 4(Aiii)), the fluorescence intensities of the biotinylated substrate increased, while the fluorescence intensities of the bare SiO_2_ substrate slightly decreased compared to those of the bare SiO_2_ substrate in Figure 4(Aii). The results show that the lateral flow increases the specific binding reactions between streptavidin and biotin molecules, while decreasing the nonspecific bindings of streptavidin molecules. These results are consistent with the results of Figure 2C, indicating the reliability of our observations. 

Figure 4B shows the real-time response curves of bare (black dot) and biotinylated (red dot) substrates during reaction with FITC-labeled streptavidin molecules. Here, the same experimental conditions as the experiments depicted in Figure 4A were used for the real-time response experiments. The experimental data of biotinylated substrates under static conditions were fitted using Equation (1), and the fitted curve is marked in a blue line. Black dots in the figure represent the fluorescence intensities of bare SiO_2_ substrates, which indicate the amount of the nonspecific adsorptions of streptavidin molecules. The streptavidin molecules specifically bound to the biotinylated substrates were almost saturated for 90 min under static conditions and increased again when we applied the lateral flows on the substrates. Note that the fluorescence intensities of bare SiO_2_ substrates showed negligible changes as a result of the lateral flow. This result indicates that the enhanced binding reactions such as those between streptavidin and biotin molecules under lateral flow conditions did not increase the nonspecific adsorptions of streptavidin molecules.

Figure 4C shows the nonspecific binding reaction curves of FITC-labeled streptavidin molecules to bare SiO_2_ substrates under static and lateral flow conditions. To obtain only nonspecifically bound streptavidin molecules, bare SiO_2_ substrates were reacted with FITC-labeled streptavidin molecules with and without lateral flows (120 mm/s) for 90 min. Black and red dots in the graph indicate experimental data under static and lateral flow conditions, respectively. The graph shows the fluorescence intensities of bare SiO_2_ substrates reacted with FITC-labeled streptavidin molecules under static conditions increase as reaction time increase. On the other hand, under lateral flow conditions, the fluorescence intensities of a bare SiO_2_ substrate reacted with FITC-labeled streptavidin molecules rapidly increase over 40 min. Then the fluorescence intensities are saturated. The saturated intensities are lower than the fluorescence intensities under static conditions. This implies that the lateral flows of a target solution hinder the nonspecific adsorptions of streptavidin molecules.

Figure 4D shows the dissociation curves of nonspecifically adsorbed FITC-labeled streptavidin molecules on bare SiO_2_ substrates under static and lateral flow conditions. For the nonspecific adsorptions of streptavidin molecules on bare SiO_2_ substrates, bare SiO_2_ substrates were incubated in 1 µg/mL streptavidin solution for 12 h at 4 °C. The incubated substrates were gently washed with a PBS solution, and the tests were conducted in PBS solution under static and lateral flow conditions for 60 min. The measured fluorescence intensities were fitted using exponential decline functions [24]. Black and red lines indicate the fitting curves of the static and lateral flow condition experiments, respectively. The graph shows that fluorescence intensities decreased significantly under lateral flow conditions, while they barely changed under static conditions. These results clearly indicate that nonspecifically adsorbed streptavidin molecules can be reduced by lateral flows. Considering that the nonspecific binding of molecules reduces the specificity of a biosensing system, the lateral flow strategy could be used to enhance the specificity of a biosensing system.

A plausible explanation for the reduced non-specific reactions could be shear forces under lateral flow conditions. To estimate the shear forces generated by lateral flows in our experiments, we first conducted the simulation of shear rate distributions around a rotating disk with a 10 nm-sized particle 15 mm from the disk’s center (Supplementary Figure S3). The shear force near the particle was estimated by multiplying the shear rate with the viscosity of water. The estimated shear force on a 10 nm-sized particle was ~2.7 pN. This corresponds to only ~1% of the reported binding force (220~460 pN) of a single streptavidin-biotin pair [25,26,27]. On the other hand, it is ~10% of the common nonspecific binding forces (20~100 pN) between streptavidin molecules and a glass substrate [27]. Since a streptavidin molecule has four biotin binding sites, the specific binding force of a single streptavidin to biotin molecules would be higher than what has been reported. Therefore, the shear forces generated by a lateral flow might significantly affect only nonspecific bindings of streptavidin molecules. These results are summarized in Table 1. Since the nonspecific bindings of target molecules could result in background noise or false signals in biosensors, our strategy could be used to enhance the selectivity of bio-sensing systems. 

### 3.3. Improved Binding Affinity under Different pH and Ionic Strength Conditions

**Figure 5:**
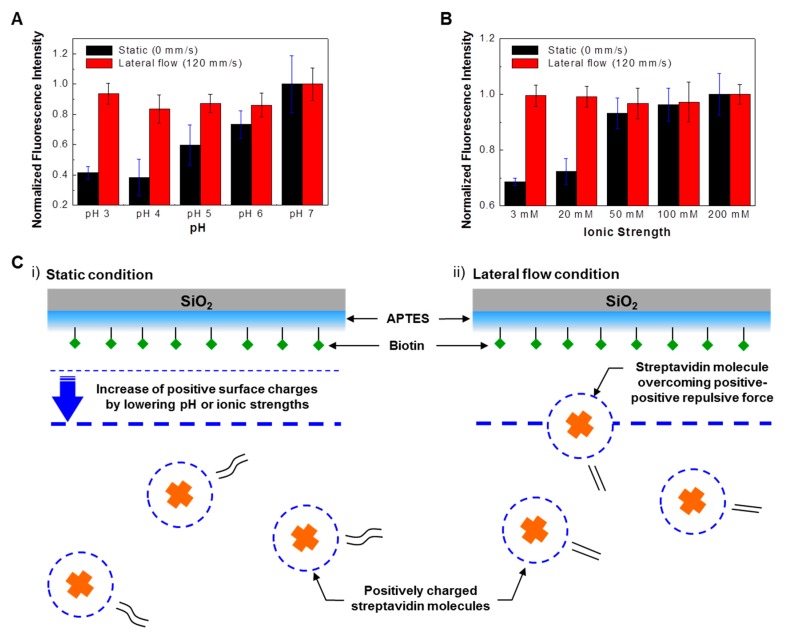
Effect of lateral flows on streptavidin-biotin reactions under various pH and ionic strength conditions: (**A**) Normalized fluorescence intensities of bound FITC-labeled streptavidin molecules on biotinylated substrates under different pH and flow speed conditions; (**B**) Normalized fluorescence intensities of bound FITC-labeled streptavidin molecules on biotinylated substrates under different ionic strength and flow speed conditions; (**C**) Schematic diagrams depicting the movements of streptavidin molecules to a biotinylated substrate under (**i**) static conditions and (**ii**) lateral flow conditions in low pH or low ionic strength solution.

Figure 5A shows the normalized fluorescence intensities of bound FITC-labeled streptavidin molecules on biotinylated substrates under different pH and flow speed conditions. In order to prepare streptavidin solutions with various pH values, we dissolved FITC-labeled streptavidin powders in citric acid-Na_2_HPO_4_ buffer solutions that had been titrated to have pH ranges from 3 to 7. The sensing experiments were conducted under static and lateral flow conditions in different pH value target solutions for 2 h. The obtained fluorescence intensities were normalized with respect to the result at pH 7. The fluorescence intensities of bound FITC-labeled streptavidin molecules under static conditions (black bars) decrease with the decrease in pH value. Previous work has shown that under low pH conditions, many sensing molecules lose their binding affinity, and sensor devices based on the sensing molecules do not work [28,29,30], which is consistent with our results. On the other hand, it should be pointed out that the fluorescence intensities under lateral flow conditions (red bars) were almost the same over the entire pH range, indicating the same amount of bound streptavidin on the biotinylated substrate. These results show that under lateral flow conditions, biotins on the substrate maintain their affinity to streptavidin, and that the biotinylated substrate can be used as a sensor device even in target solutions with very low pH. 

We also performed a similar experiment with FITC-labeled streptavidin binding onto biotinylated substrates under different ionic strength and flow speed conditions (Figure 5B). In order to prepare streptavidin solutions with different ionic strengths, 1 M NaCl solutions were added to sodium acetate buffer solutions (pH 5) such that they had ionic strengths ranging from 3 mM to 200 mM. Then, FITC-labeled streptavidin powders were dissolved in the solutions to result in streptavidin concentrations of 1 ng/mL. The fluorescence intensities of bound FITC-labeled streptavidin molecules under static conditions (black bars) decreased with a decrease in ionic strength. Presumably, at a low ionic strength, the binding affinity of biotin onto streptavidin became weaker, as reported previously [31]. Interestingly, fluorescence intensities under lateral flow conditions (red bars) are maintained regardless of ionic strength changes. These results imply that the binding affinity of streptavidin-biotin reactions is maintained by the lateral flows of the target solution even in low ionic strength conditions. Such an improved binding affinity under lateral flow conditions has not been reported before.

Figure 5C shows schematic diagrams depicting a plausible explanation of the improved binding affinity observed in our experiments under lateral flow conditions. It shows the movement of streptavidin molecules to a biotinylated biosensor under (i) static and (ii) lateral flow conditions in low pH or low ionic strength solutions. Previous work has shown that the decrease in binding affinity between interacting molecules in low pH or low ionic strength solutions can be explained by the net electrical charge of molecules [32,33]. When two interacting molecules with the same electrical charge approach each other, long-range electrostatic repulsive forces between the molecules increase, which obstruct binding interactions between the two molecules. The net electrical charges of streptavidin molecules and biotinylated SiO_2_ substrates were reported to have positive values below a pH of 6 [32,34,35]. These positive charges increase as pH or ionic strength decreases, enhancing the repulsive forces between the streptavidin molecules and biotinylated surfaces. Therefore, binding reactions between streptavidin and biotin molecules can be attenuated by lowering the pH or ionic strengths of a liquid medium (Figure 5(Ci)).

On the other hand, under lateral flow conditions, as shown in Figure 5(Cii), the lateral flows increase the molecular speed of streptavidin molecules as well as the mass transfer rates of the molecules to the biotinylated surface. Presumably, the increased molecular speeds and mass transfer rates could help in overcoming the electrostatic repulsive forces between the streptavidin molecules and the biotinylated substrate and enhance the probability of the presence of streptavidin molecules near the biotinylated surface. This could induce an increase in effective collisions between streptavidin and biotin molecules under lateral flow conditions, allowing the binding rate of streptavidin-biotin molecules to be maintained even under low pH and ionic conditions. We also performed experiments investigating the effects of a lateral flow on the binding reactions between IL-13 antigens and IL-13 antibody-coated substrates at various pH conditions and found that the binding reactions of immune proteins could also be enhanced under low pH conditions by lateral flow in the target solution (Supplementary Figure S4). In many biosensing systems, the changes of the environmental conditions of target solutions hinder the quantitative detections of target molecules. Thus, our lateral flow strategy could also be a powerful tool for developing a quantitative biosensing system without worries about environmental conditions.

## 4. Conclusions

In conclusion, we report a lateral flow strategy that can enhance the reaction speed, selectivity and sensitivity of a conventional biosensor even under low pH and ionic strength conditions. As a proof of concept, we utilized streptavidin-biotin reactions and IL-13 antigen-antibody reactions to analyze the effects of the lateral flows on binding kinetics between target molecules and a biosensing substrate. We found that the binding speeds and affinity of target molecules for their sensing molecules on the sensing substrates were significantly enhanced by lateral flow in the target solution. Moreover, the lateral flow generated shear forces on the sensing substrate, which removed non-specifically bound molecules from the substrate. This result implies that the lateral flow strategy could also be used to enhance the selectivity of a biosensing system. Finally, we observed enhanced binding affinity between target molecules and sensing molecules even under low pH and ionic strength conditions, which could be important in quantitative detection of target molecules in real samples. Since the lateral flow strategy can easily improve the performance of biosensors simply by generating the lateral flows of a target solution on a sensor surface, our strategy could be a useful tool for versatile bio and medical applications.

## Figures and Tables

**Table 1:** Comparison of calculated shear force with specific and nonspecific binding forces.

	Specific Binding Force of Streptavidin-Biotin	Nonspecific Binding Force of Streptavidin	Calculated Shear Force on a Rotating Disk (120 mm/s)
Force (pN)	220~460	20~100	2.7
$\frac{Shear\;force}{Binding\;force}\times 100$ (%)	0.6~1.2	2.7~13.5	-

## Supplementary Materials

The following are available online at http://www.mdpi.com/1424-8220/18/5/1507/s1, Figure S1: Simulation of fluidic flows generated by the rotation of a rotating disk with 150 rpm, Figure S2: Simulation results of bound target molecules on sensing substrates located at 0, 3, 9 and 15 mm of a rotating disk, Figure S3: Simulation results of shear rates, Figure S4: Effect of different flow conditions on IL-13 antibody-antigen reactions under various pH conditions.
