# Supplementary material for: High-Speed Lateral Flow Strategy for a Fast Biosensing with an Improved Selectivity and Binding Affinity
Source: Sensors (Basel). 2018 May 10;18(5):1507. doi: 10.3390/s18051507 (PMC5982462; doi:10.3390/s18051507)
Supplement: Supplementary file 1 [file sensors-18-01507-s001.pdf]

[Supplementary Material]

High-speed lateral flow strategy for a fast biosensing with an improved selectivity and binding affinity

Dong Guk Cho[1], *Haneul Yoo*[1], *Haein Lee*[2], *Yeol Kyo Choi*[2], *Minju Lee*[1], *Dong June Ahn*[*,2]

and Seunghun Hong[*,1]

[1] Department of Physics and Astronomy and Institute of Applied Physics, Seoul National University, Seoul 151-747, Korea

[2] Department of Chemical & Biological Engineering, and KU-KIST Graduate School of Converging Science & Technology, Korea University, Seoul 02841, Korea.

Figure S1

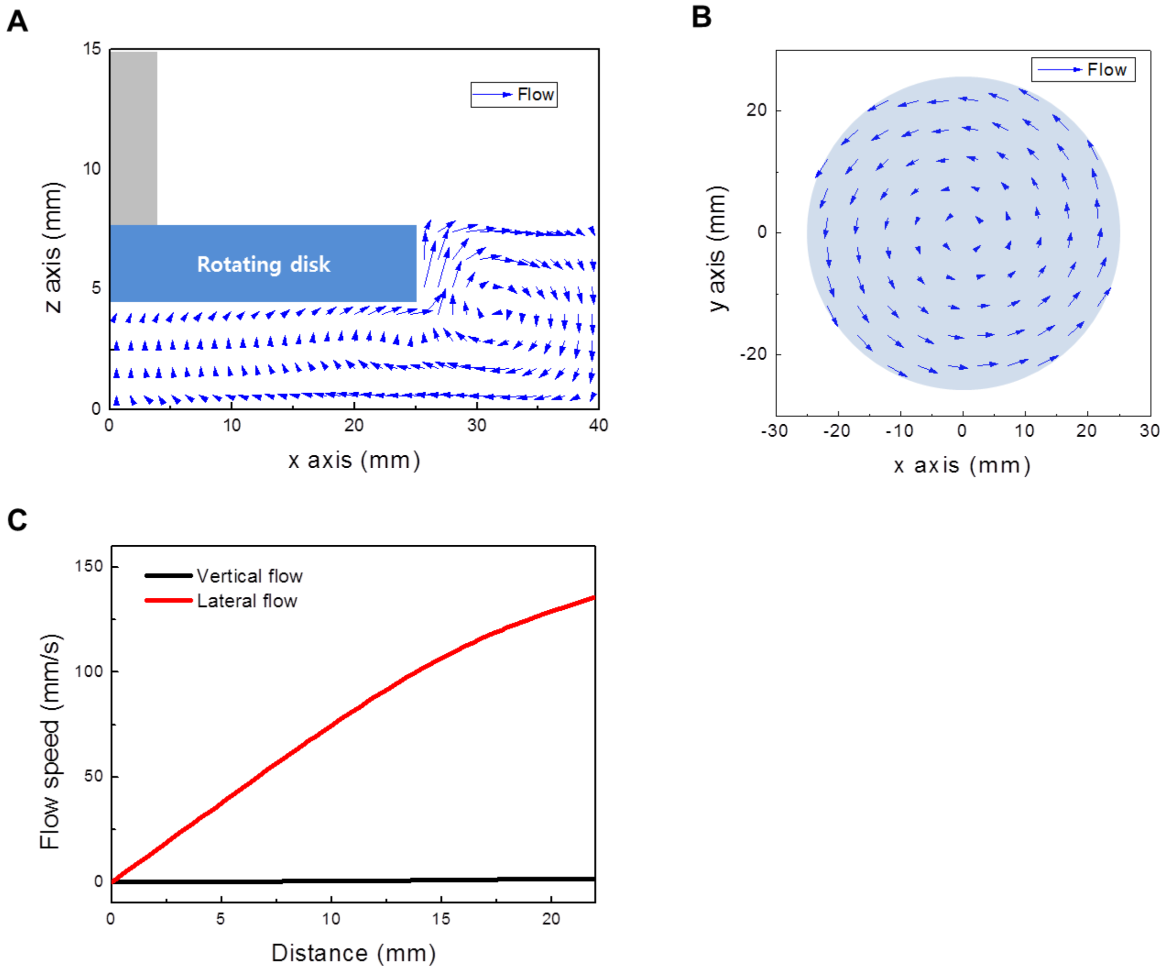

Figure S1. Simulation of fluidic flows generated by the rotation of a rotating disk with 150 rpm: (A) Cross section view of fluidic flows; (B) Lateral fluidic flows on the rotating disk; (C) Vertical and lateral flow speeds with distances from the center of the disk.

Figure S2

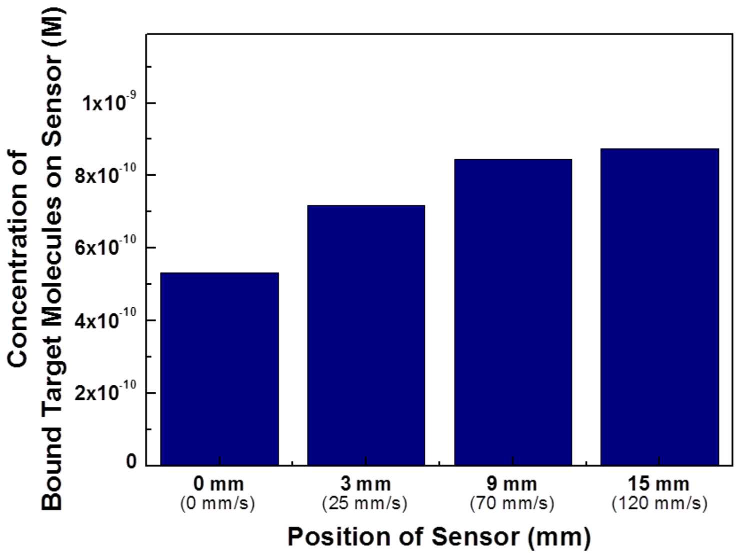

Figure S2. Simulation results of bound target molecules on sensing substrates located at 0, 3, 9 and 15 mm of a rotating disk. For the simulation, a commercial finite element method package (COMSOL Multiphysics) was used for the simulation of binding kinetics between a sensing substrate and target molecules. Target molecules were initially dispersed equally over a target solution. *The diffusion constant of target molecules* and *a binding constant K_D between the sensing substrate and target molecules* was assumed as 10^{-10} m/s^2 and 10^{-9} M, respectively. The reaction time was 2 hours. Considering the turbulent flows of a target solution, 15 mm of the rotating disk was selected as the outermost position where the sensing substrate can be located. The result shows that the difference of bound target molecules between the sensing substrates at 0 and 3 mm are higher than the sensing substrate at 9 and 15 mm, which indicate the increase rate of bound target molecules is higher at the inner region of the disk than the outer region of the disk.

Figure S3

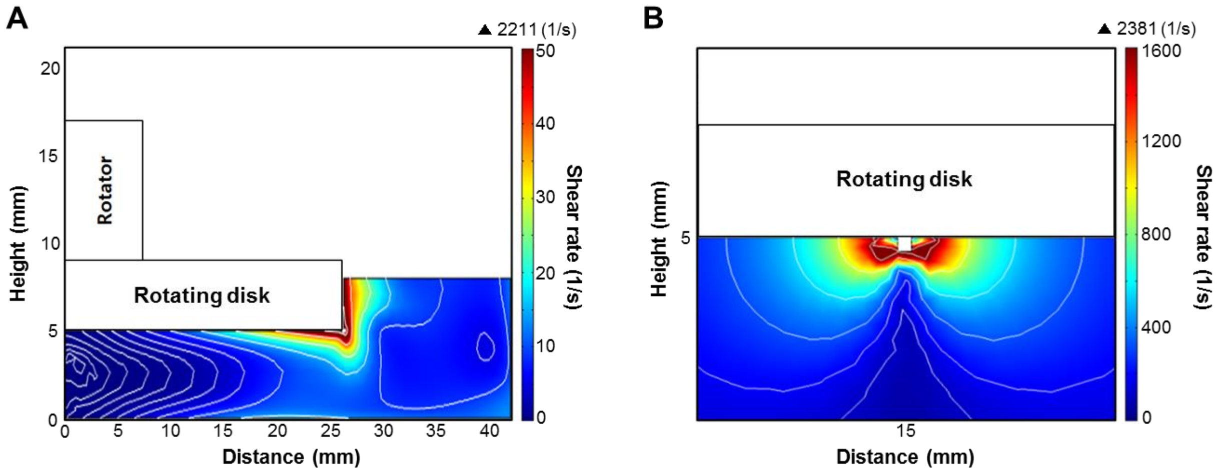

Figure S3. Simulation results of shear rates. The rotating speed of a rotating disk was set to 150 rpm: (A) Shear rate distribution in a reaction chamber; (B) Shear rate distribution on a 10 nm-sized particle attached on 15 mm from the center of the disk.

Figure S4

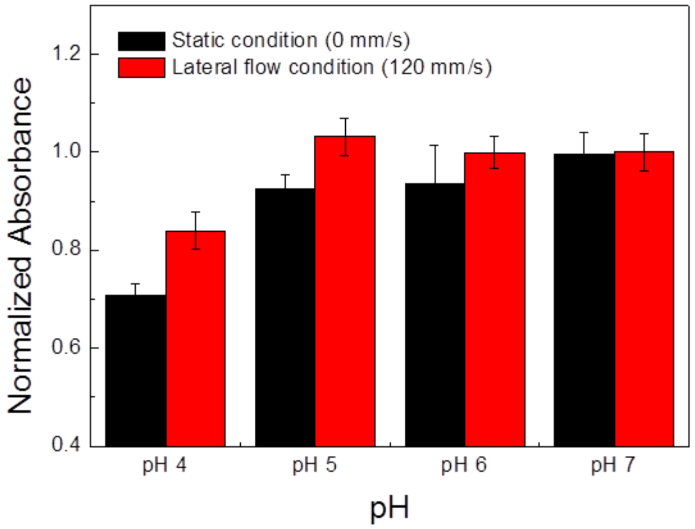

Figure S4. Effect of different flow conditions on IL-13 antibody-antigen reactions under various pH conditions
